# INTERBED: internet-based guided self-help for overweight and obese patients with full or subsyndromal binge eating disorder. A multicenter randomized controlled trial

**DOI:** 10.1186/1745-6215-13-220

**Published:** 2012-11-21

**Authors:** Martina de Zwaan, Stephan Herpertz, Stephan Zipfel, Brunna Tuschen-Caffier, Hans-Christoph Friederich, Frauke Schmidt, Olaf Gefeller, Andreas Mayr, Tony Lam, Carmen Schade-Brittinger, Anja Hilbert

**Affiliations:** 1 Department of Psychosomatic Medicine and Psychotherapy, Hannover Medical School, Carl-Neuberg-Strasse 1, D-30625, Hannover, Germany; 2 Department of Psychosomatic Medicine and Psychotherapy, LWL-University, Ruhr-University Bochum, Alexandrinenstrasse 1-3, D-44791, Bochum, Germany; 3 Psychosomatic Medicine and Psychotherapy, Medical University Hospital Tübingen, Osianderstrasse 5, D-72076, Tübingen, Germany; 4 Department of Clinical Psychology and Psychotherapy, University of Freiburg, Engelbergerstrasse 41, D-79085, Freiburg, Germany; 5 Department of General Internal Medicine and Psychosomatics, Medical University Hospital Heidelberg, Im Neuenheimer Feld 410, D-69120, Heidelberg, Germany; 6 Department of Psychosomatic Medicine and Psychotherapy, Medical University Hospital Erlangen, Schwabachanlage 6, D-91054, Erlangen, Germany; 7 Department of Medical Informatics, Biometry and Epidemiology, University of Erlangen-Nuremberg, Waldstrasse 6, D-91054, Erlangen, Germany; 8 NetUnion sarl, Ave de Villamont 19, CH-1005, Lausanne, Switzerland; 9 Coordinating Centre for Clinical Trials (KKS), Philipps-University Marburg, Karl-von-Frisch-Strasse 4, Marburg, D-35043, Marburg, Germany; 10 Department of Medical Psychology and Medical Sociology, Integrated Research and Treatment Center Adiposity Diseases, University of Leipzig Medical Center, Stephanstrasse 9C, D-04103, Leipzig, Germany

**Keywords:** Binge eating disorder, Internet-based self-help, Cognitive-behavioral therapy, Noninferiority trial

## Abstract

**Background:**

Binge eating disorder (BED) is a prevalent clinical eating disorder associated with increased psychopathology, psychiatric comorbidity, overweight and obesity, and increased health care costs. Since its inclusion in the DSM-IV, a few randomized controlled trials (RCTs) have suggested efficacy of book-based self-help interventions in the treatment of this disorder. However, evidence from larger RCTs is needed. Delivery of self-help through new technologies such as the internet should be investigated in particular, as these approaches have the potential to be more interactive and thus more attractive to patients than book-based approaches. This study will evaluate the efficacy of an internet-based guided self-help program (GSH-I) and cognitive-behavioral therapy (CBT), which has been proven in several studies to be the gold standard treatment for BED, in a prospective multicenter randomized trial.

**Methods:**

The study assumes the noninferiority of GSH-I compared to CBT. Both treatments lasted 4 months, and maintenance of outcome will be assessed 6 and 18 months after the end of treatment. A total of 175 patients with BED and a body mass index between 27 and 40 kg/m^2^ were randomized at 7 centers in Germany and Switzerland. A 20% attrition rate was assumed. As in most BED treatment trials, the difference in the number of binge eating days over the past 28 days is the primary outcome variable. Secondary outcome measures include the specific eating disorder psychopathology, general psychopathology, body weight, quality of life, and self-esteem. Predictors and moderators of treatment outcome will be determined, and the cost-effectiveness of both treatment conditions will be evaluated.

**Results:**

The methodology for the INTERBED study has been detailed.

**Conclusions:**

Although there is evidence that CBT is the first-line treatment for BED, it is not widely available. As BED is still a recent diagnostic category, many cases likely remain undiagnosed, and a large number of patients either receive delayed treatment or never get adequate treatment. A multicenter efficacy trial will give insight into the efficacy of a new internet-based guided self-help program and will allow a direct comparison to the evidence-based gold standard treatment of CBT in Germany.

**Trial Registration:**

Current Controlled Trials ISRCTN40484777

German Clinical Trial Register DRKS00000409

## Background

Binge eating disorder (BED) is a research diagnosis in the *Diagnostic and Statistical Manual of Mental Disorders*, *Fourth Edition* (DSM-IV) [1]. BED is defined by recurrent binge eating episodes that occur, in contrast with those in bulimia nervosa, in the absence of inappropriate weight control behaviors (for example, purging). A series of characteristics are associated with binge eating, such as rapid consumption of food, eating until uncomfortably full, and marked distress regarding the behavior. For a BED diagnosis, binge eating episodes must have occurred at least twice weekly over a period of 6 months.

International experts for eating disorders agree that BED is a valid eating disorder diagnosis and should be included in DSM-5 as an official diagnosis [2]. BED is the most prevalent eating disorder, affecting 2% to 5% of the general population, and both genders appear to be equally affected [3]. The disorder is associated with substantial medical [4] and psychological comorbidities [5]. Furthermore, overweight and obesity are common in patients with BED. Up to 30% of participants in weight loss programs meet criteria for BED [6], and higher levels of binge eating have been linked to overweight and obesity [7]. Obese patients with BED display marked eating disorder psychopathology and comorbidity with other psychiatric disorders. Given the associated comorbid somatic and mental sequelae, BED is argued to be a disorder of clinical significance causing huge costs for the medical system [8].

According to current meta-analyses and clinical treatment guidelines, cognitive-behavioral therapy (CBT) is regarded as the first-line specialty treatment for BED [9-13]. Controlled studies of CBT generally report substantial reductions in binge eating and in most associated problems such as comorbid psychopathology and impaired quality of life [13].

Although CBT is the gold standard treatment for BED patients, this intervention is not offered areawide, leading to delayed delivery of adequate treatment. An alternative to classic face-to-face CBT and a potential means by which to disseminate adequate treatment for eating disorders is guided or pure self-help for patients with BED. So far only a few open studies or RCTs have evaluated self-help interventions in BED [7,14-20]. Nearly all of them used book-based self-help with manuals detailing CBT for binge eating, primarily the book *Overcoming Binge Eating*[21]. One study used a CD-ROM-based self-help intervention [18]. Results of single studies, meta-analyses, and systematic reviews [13,22-24] have shown that guided self-help is superior to waiting list in patients with BED. Patients using self-help modalities did better than controls in reducing days with objective binge eating episodes (OBEs), in reducing hours spent binge eating, and in improving the specific eating disorder psychopathology [15]. However, the evidence regarding guided self-help is limited to a small number of studies, and further, larger randomized-controlled studies (RCTs) are needed.

Several experts have outlined potential advantages of guided self-help treatments. (1) They allow evidence-based treatments to be offered with minimum delay. (2) They are popular and acceptable to many patients. (3) They can be offered at low cost. (4) They respect patients’ privacy and avoid their embarrassment about needing psychotherapy. (5) They allow patients to work at their own pace, which is particularly important for highly anxious or depressed patients who have difficulty to focus during a session with a therapist. (6) They allow patients to renew or update treatment as often as they wish and at no extra cost. (7) They could be appropriate for less severe conditions in primary care delivered by trained nonspecialists or could be the first step in patients’ search for a more comprehensive treatment.

Only one study directly compared guided self-help with face-to-face psychotherapy [19]. In this trial, interpersonal psychotherapy (IPT; n = 75) and guided self-help based on CBT using a book-based format (n = 66) were equally effective, with 4-week abstinence rates from OBEs in more than 60% of the patients that were maintained over a follow-up period of 4 years [25]. However, IPT was more successful than self-help in retaining patients in the trial. Moderator analyses provided evidence for a specificity of treatment effects; for example, a high baseline binge eating frequency had a negative impact on remission rates in the guided self-help condition, but not in the IPT condition.

Until recently, little has been done on technology-enhanced delivery of CBT-based interventions for BED. In a RCT comparing the efficacy of a 10-week CD-ROM intervention, a group CBT, and a waiting list, there were comparable reductions in days with OBEs in the group CBT and in the CD-ROM condition, with better results in the two active intervention groups compared to the waiting list [18]. However, the conclusions of this study were limited by a high rate of dropout. At present, there is only one internet-based BED treatment study comparing a guided self-help approach with a waiting list [20]. Seventy-four women were randomized to either a 6-month online program with a 6-month follow-up or a 6-month waiting list. Guidance consisted of regular e-mail contact with a coach during the whole intervention. The number of OBEs and eating disorder psychopathology significantly improved after the internet self-help treatment intervention. Improvements were maintained at 6-month follow-up. Overall, a transfer of CBT-based self-help techniques to the internet was well-accepted by patients and showed positive results for eating disorder psychopathology. For many participants, it was their first eating disorder treatment. However, internet-based, guided self-help has not yet been directly compared with standard face-to-face CBT.

An additional important question is for whom the face-to-face and self-help modalities of CBT work [10]. Evidence from the planned treatment trial would allow specifying how to adequately match patients with treatments. A comparison of previous studies did not show any clear difference in moderators for both face-to-face and self-help modalities of CBT. More severe eating disorder psychopathology and general psychopathology inconsistently predicted poor treatment outcome in both modalities [26]. From a stepped care approach, as advanced by the National Institute for Clinical Excellence guidelines [11], patients with more severe psychopathology should benefit more from face-to-face CBT, whereas internet-based guided self-help should be sufficient for patients with low psychopathology. Further variables with at least some evidence for predictive effects on treatment outcome are psychiatric comorbidity, self-esteem, quality of life, age at onset of the eating disorder or of overweight, and patient expectation and motivation (for a summary, see [27]).

The main goal of internet-based guided self-help for overweight and obese patients with binge eating disorder (INTERBED) is to compare the short- and long-term outcomes of two treatments for adult patients with BED: internet-based guided self-help treatment (GSH-I) and CBT in an individual setting. In addition, we will investigate predictors and moderators of treatment outcome. Finally, we will evaluate the cost-effectiveness of both treatments.

## Methods

### General design aspects

INTERBED is a multicenter, randomized, noninferiority trial with two parallel arms designed to evaluate, in an independent and blinded study, the efficacy of GSH-I and CBT (principal investigator (PI): MdZ; co-PI: AH). The treatment phase will last 4 months per participant. A maximum of 6 weeks can be added in case of longer therapy intervals due to factors such as illness or vacation. Study enrollment was started in August 2010 and finished in March 2012. After randomization, baseline assessment was conducted (T0) prior to the start of therapy, and within 2 weeks participants started treatment in either the GSH-I or the CBT arm.

Participants then received 20 individual face-to-face treatment sessions with a therapist (CBT) or had 17 to 18 e-mail contacts and 2 personal contacts with a coach (GSH-I) over a period of 4 months. The number of days on which OBEs occurred were assessed at midtreatment, at month 2 (GSH-I), or after 10 therapy sessions (CBT) (T1), as well as after the completion of treatment (T2). Both treatments lasted 4 months, and maintenance of outcome will be assessed 6 months and 18 months after the end of treatment (T3 and T4).

### Study centers and participants

The study is being conducted at seven trial sites, all of which are running well-established outpatient clinics. All participants have been registered at the outpatient clinics of the trial sites. Participating centers are the Departments of Psychosomatic Medicine and Psychotherapy of the Universities of Bochum (PI: SH), Erlangen-Nuremberg (PI: MdZ), Heidelberg (PI: HCF), and Tübingen (PI: SZ); the Institutes of Clinical Psychology of the Universities of Freiburg (PI: BTC) and Fribourg/Switzerland (PI: AH); and the Integrated Research and Treatment Center Adiposity Diseases of the University of Leipzig (PI: AH). The study was advertised in the respective catchment areas of the participating institutions in order to recruit a sufficient number of participants.

A minimum of 70 participants finishing the study is required for each study arm. With an expected dropout rate of 20%, 175 participants needed to be recruited. Overall, 178 participants were enrolled, and 89 were randomized to each treatment condition. Between 10 and 36 participants were randomized at each of the 7 trial sites.

To be included in the study, participants needed to be 18 years of age or older, have a body mass index (BMI) between 27 and 40 kg/m^2^, meet diagnostic criteria for BED according to DSM-IV-TR or subsyndromal BED, and have available internet access. To ensure generalization of study results, exclusion criteria were kept to a minimum and are listed below. Overall, 586 individuals were screened for participation, of whom 408 did not meet inclusion criteria. The most common reasons for nonparticipation were BMI ≥40 kg/m^2^ (n = 105), BMI <27 kg/m^2^ (n = 65), ongoing psychotherapy (n = 40), not meeting diagnostic criteria for BED or subsyndromal BED (n = 57), meeting criteria for bulimia nervosa (n = 14), and lack of interest in the study (n = 57).

### Inclusion criteria

•Diagnostic criteria for BED according to DSM-IV, or

•Subsyndromal BED: Patients have to meet the criteria for OBEs but can lack one of the other DSM-IV criteria (frequency of less than 2 days with OBEs in 6 months, no marked distress, or presence of only 2 instead of 3 of the 5 associated criteria)

•Age 18 years or older

•27 ≤ BMI < 40 kg/m^2^

•Written informed consent of the patient

### Exclusion criteria

•Current bulimia nervosa

•Current substance abuse

•Current suicidal ideation

•Psychotic disorder

•Bipolar disorder

•Serious unstable medical problems or conditions (for example, type 1 diabetes mellitus or thyroid problems) that influence weight or eating

•Ongoing psychotherapy

•Current intake of antipsychotic or weight-affecting drugs

•Pregnancy or lactation

### Interventions

As mentioned above, therapy was given over a period of 4 months (with a maximum of 6 additional weeks). Each therapist was responsible for both treatments, as a coach in the GSH-I arm and as a therapist in the CBT arm. Prior to the start of treatment, therapists received training for the GSH-I (provided by TL) as well as training for the CBT program (provided by AH).

#### Experimental intervention 1: internet-based guided self-help (GSH-I)

For this intervention, the Self-Help Guide (Copyright © NetUnion & University Hospital of Geneva (HUG)) was used. This program is based on an online program for bulimia nervosa following CBT principles that was developed in the European Research Program SALUT by HUG and NetUnion and adapted to specifically address BED [20]. The program was translated from French into German by the INTERBED study team. It consists of 11 sequential modules which are delivered within 4 months (see Table 1). Participants work through the modules sequentially. After predefined time intervals, the next module is made accessible to the participants by the coach (see Table 2).

**Table 1:** **Modules of the internetbased selfhelp**[20]

**Modules**	**Key exercises**/**examples**
Module 1: Preparing for change (motivation module)	· Understanding the mechanisms that perpetuate the eating problem
	· Looking at self-esteem, mental attitude, and interpersonal relationships
	· Advantages and disadvantages of the current behavior
	· Imagining the future after successfully finishing the program
	· Conditions for change
Module 2: Observing yourself	· My food diary
	· Food diary summary
	· Sabine’s day
	· Sabine’s food diary
Module 3: Understanding and trusting yourself	· The possibility of eating for pleasure
	· Food- and emotion-related triggers for compulsive eating
Module 4: Finding your own rhythm (Goal: A healthy meal pattern)	· Eating regularly and according to my own rhythm
	· Finding my own preferences
	· Giving yourself time
Module 5: Building up your strategies	· How to prevent compulsive eating
	· Building my own strategy list
Module 6: Physical activities	· How to get started?
	· What is an “activity break”?
Module 7: Identifying and solving your problems	· Learning how to solve a problem in separate steps
Module 8: Self-assertion	· Making a place for yourself in the world
	· Using new assertiveness techniques
Module 9: Handling your emotions	· Automatic thoughts that trigger these emotions
Module 10: Changing the way you think	· Becoming more aware of certain cognitive distortions
	· Changing automatic thoughts into realistic thoughts
	· Applying these techniques to thoughts concerning food and your figure
Module 11: Continuing on your way	· Remembering what you have learned and preventing relapses
	· Using some tools in case of a misstep

**Table 2:** **Frequency of therapy sessions**/**e**-**mail contacts in the GSH**-**I and CBT conditions**

		**Month 1**	**Month 2**	**Month 3**	**Month 4**
GSH-I Duration: 4 months 17–18 e-mails 2 face-to-face sessions	Frequency of contact	One face-to face contact with patient	One weekly e-mail contact with patient	One weekly e-mail contact with patient	One face-to face contact with patient
		One weekly e-mail contact with patient			One weekly e-mail contact with patient
	Time flow of the intervention modules	Module 1-3	Module 3-6	Module 6-9	Module 9-11
		**Month 1**	**Month 2**	**Month 3**	**Month 4**
Individual CBT Duration: 4 months 20 face-to-face sessions	Frequency of contact	Twice weekly face-to-face sessions with patient	One weekly face-to-face session with patient	One weekly face-to-face session with patient	One weekly face-to-face session with patient
	Time flow of the intervention phases	Phase 1-2	Phase 2	Phase 2	Phase 3

Each module combines psychoeducation and behavioral interventions and exercises that participants complete directly in the program. A self-monitoring diary is introduced from the second module on and is used throughout the duration of the treatment. Automatic feedback generated by the program provides an objective view of frequency and development of participants’ behavior. Two fictitious characters illustrate all exercises and techniques. They demonstrate how to complete exercises, explain difficulties, and give specific examples to the participants. Some of the modules (that is, relaxation and mindfulness) have audiotape files attached to them for the participants to listen to.

Coaches can monitor participants’ progress and review completed exercises, diary entries, and automatic feedback charts to ensure the correct use of the program. Participants are instructed to contact their coach at least once weekly by e-mail, and they receive feedback by e-mail once weekly on a fixed day from the coach. Coaches provide support and encouragement and reinforce program participation through motivational messages, as outlined in a coaches’ manual (written by TL and FS) outlining the structure and content of the e-mails, including sample e-mails, and giving instructions on how to handle unexpected emergencies (for example, suicidality). Adherence to the content and structure of the e-mails is supervised by an experienced psychologist at the trial site in Erlangen-Nuremberg, who gives written feedback to the coaches.

If participants do not enter the program or do not write any e-mails for 3 weeks, the coaches call them on the telephone to increase their motivation. After 4 weeks without any connection, the participants’ program access is canceled and participants are considered study dropouts.

Coaches and participants meet in person twice for 90 minutes before the beginning and after the end of treatment. Both sessions are audiotaped. During the first face-to-face session, the coaches explain the program’s rationale to ensure the correct use of the internet program. During the second face-to-face session, coaches and participants briefly discuss treatment success and, if necessary, further treatment options. After the end of the treatment phase, the coach cancels participants’ access to the program; however, all modules are available for download in PDF format, and participants are advised to print out the self-help material to have the material available after the end of the guided treatment phase.

To ensure confidentiality and data protection, the online program uses a password-protected server, which is located at NetUnion, Lausanne, Switzerland. Participants receive a pseudonym and a password to access the online program. For security reasons, they have to change their password at first connection. An integrated messaging system enables secured message exchange between coaches and participants. E-mail addresses are protected by one-way encryption. The website meets Health on the Net Foundation (HON) quality and ethics standards (http://www.hon.ch/).

#### Experimental intervention 2: cognitive-behavioral therapy (CBT)

For this individual intervention, the existing manual in German, “*Binge Eating and Obesity*: *CognitiveBehavioral Therapy Manual for Binge Eating Disorder*” by Hilbert and Tuschen-Caffier [28] is used. The manual comprises the following phases: (1) initial treatment phase for motivational enhancement; (2) intensive treatment phase, including modules on eating behavior, body image, and stress; and (3) self-management phase for relapse prevention (Table 3).

**Table 3:** **Modular cognitive**-**behavioral group therapy for binge eating disorder**^**a**^

	**Therapeutic goals**	**Therapeutic interventions and techniques**
Initial treatment phase (sessions 1 to 3)	· Motivational enhancement	· Psychoeducation
		· Self-monitoring of food intake
		· Development of an individual maintenance model
		· Goal-setting
		· Cognitive interventions for motivation
Intensive treatment phase (sessions 4 to 17)	· Normalizing eating behavior	Eating Behavior Module
	· Identification and modification of dysfunctional thoughts and schemata	· Nutritional management
	· Acquisition of new skills	· Hunger and satiety perception training
	· Establishment of regular physical activity	· Cue exposure
		· Hedonics exercise
		· Cognitive interventions for negative schemata related to eating behavior
		Body Image Module
		· Body image diary
		· Body image exposure
		· Exposure to avoided body-related situations
		· Body hedonics exercise
		· Shaping of regular physical activity
		· Cognitive interventions for negative body-related schemata
		Stress Module
		· Stimulus and response control
		· Stress management techniques
		· Affect regulation techniques
		· Interpersonal problem-solving
		· Social competence training
		· Cognitive interventions for further relevant negative schemata
		· Exposure in case of further significant anxiety and avoidance
		· Homework and practice
		General techniques used in all modules
		· Psychoeducation
		· Self-monitoring
		· Goal-setting
		· Self-reinforcement
Self-management phase (sessions 18 to 20)	· Patients become their own therapists	· Development of realistic expectations concerning potential setbacks of binge eating
	· Maintaining progress in the future	· Development of relapse prevention strategies
	· Relapse prevention	

^a^From Hilbert and Tuschen-Caffier [28].

**Figure 1:**
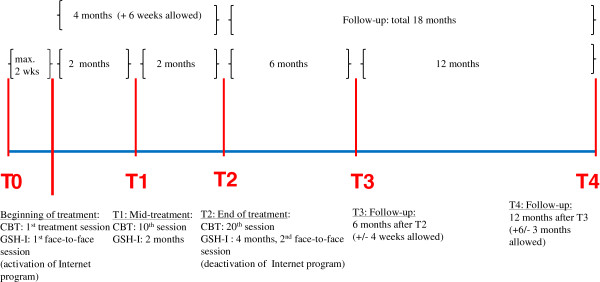
Time points of measurement in the INTERBED study.

Treatment modules are selected according to a participant’s individual symptomatology and resources. The individual outpatient treatment comprises 20 individual sessions and lasts 4 months. Participants receive therapy twice weekly for the first month and once weekly from month 2 to month 4. The therapy sessions are held at the individual trial sites. The frequency of the sessions is shown in Figure 1.

To evaluate the adherence to the CBT manual, a number of measures are assessed. First, to systematically assess characteristics of form and content as well as aspects of adherence of treatment, therapists complete structured session reports after each treatment session. The session reports give information regarding whether the treatment session was conducted as scheduled or was canceled by the participant, as well as whether the session began on time or was delayed. Furthermore, the session reports show the duration of the session, the primary and secondary foci of interventions (for example, self-monitoring), the use of manualized treatment material, reasons for nonadherence to the agenda, accomplishment of homework by the participant, and the cooperation of the participant as rated by the therapist. An individual study therapy is considered to have been conducted according to the manual if a participant attends at least 12 of 20 treatment sessions.

Second, all CBT sessions are audiotaped if participants give their consent. One of four consecutive audiotapes of each participant is randomly chosen and checked with regard to manual adherence using a checklist on content, the material worked on, and formal characteristics (for example, duration of the session). Nearly all participants gave their written informed consent for audiotaping of their therapy. Interrater reliability of the adherence coding will be determined.

### Objectives and hypotheses

The objectives of the study are to assess the short- and long-term efficacy of internet GSH-I compared to individual CBT with respect to the number of days with OBEs and with respect to secondary outcomes of treatment, to investigate moderators and predictors of therapeutic change, and to determine cost-effectiveness.

In accordance with the primary objective, the GSH-I arm is expected to show no significant inferiority regarding the decrease in the number of days with OBEs compared to the CBT condition. The study is designed as a noninferiority trial with a noninferiority margin of 1 day with OBEs (d) in favor of CBT. Given a lack of research supporting an evidence-based noninferiority margin at the time of study planning, this margin has been agreed upon in discussions with clinical experts. The null hypothesis tested in the confirmatory analysis is (H0) Δ GSH-I < Δ CBT-d with the alternative hypothesis of (H1) Δ GSH-I ≥ Δ CBT-d. It is also expected that there will be no significant difference in secondary outcomes between participants of the GSH-I and the CBT condition.

### Primary and secondary outcomes

The primary outcome will be the difference in the number of days with OBEs over the past 28 days. The diagnostic criteria for BED focus on days per week with OBEs rather than on individual episodes, the rationale being that, in the absence of purging behavior, BED participants may have more difficulty than patients with bulimia nervosa recalling discrete binge eating episodes. Number of days with OBEs is measured using the German version of the Eating Disorder Examination (EDE) [29,30], a semistructured interview which is regarded as the gold standard assessment of eating disorder psychopathology. Comparisons will be made between baseline (randomization, T0), midtreatment (T1), and the end of treatment (T2). In addition, maintenance of treatment outcome will be assessed 6 and 18 months after treatment completion in the two intervention groups (T3 and T4).

Secondary outcome measures are the associated eating-related psychopathology measured using the EDE subscales (restraint, eating concerns, shape and weight concerns) [29,30] and the Dutch Eating Behavior Questionnaire (DEBQ) [31,32], psychiatric comorbidity assessed using the Structured Clinical Interview for DSM-IV diagnoses (SCID-I) [33], severity of depression measured using the Beck Depression Inventory II (BDI-II) [34,35], and self-esteem measured using the Rosenberg Self-Esteem Scale (RSE) [36,37]. Quality of life will be measured using the Impact of Weight on Quality of Life Scale-Lite (IWQOL-Lite) [38,39], physical activity will be measured using the International Physical Activity Questionnaire (IPAQ) [40], and BMI will be calculated from measured weight and height (kg/m^2^).

For all self-report instruments used in the study, sufficient psychometric properties of the German versions have been demonstrated. Measurement time points are shown in Figure 1, and assessment of secondary outcome measures are given in Table 4.

**Table 4:** **Assessment of secondary outcome measures in the INTERBED study**^**a**^

	**Baseline**	**Midtreatment**	**End of treatment**	**Follow**-**up**
		**2 months after randomization**	**4 months after randomization**	**10 and 22 months after randomization**
EDE	X	X	X	X
DEBQ	X	X	X	X
SCID-I	X			X
BDI-II	X	X	X	X
RSE	X	X	X	X
IWQOL	X	X	X	X
IPAQ	X	X	X	X
BMI	X	X	X	X

^a^BDI-II = Beck Depression Inventory, DEBQ = Dutch Eating Behavior Questionnaire, EDE = Eating Disorder Examination, IPAQ = International Physical Activity Questionnaire, IWQOL = Impact of Weight on Quality of Life–Lite, RSE = Rosenberg Self-Esteem Scale, SCID-I = Structured Clinical Interview for DSM-IV, Axis I Diagnoses.

### Predictor variables

Moderator and nonspecific predictor variables are assessed pretreatment and include the severity of eating disorder and general psychopathology, which are operationalized through the EDE total score and the BDI-II. In addition, as nonspecific predictors (that is, pretreatment measures that may have a main effect on outcome but no interactive effect with treatment), frequency of binge eating, specific aspects of eating disorder psychopathology, psychiatric comorbidity, age at onset of eating disorder and overweight, self-esteem, and quality of life are assessed (EDE, DEBQ, SCID-I, RSE, and IWQOL). In addition, patient expectations and motivation are assessed using visual analogue scales (see [41]).

The process variables include variables previously shown to promote changes in face-to-face CBT such as a rapid reduction of binge eating during the early treatment phase, a decrease in weight concerns, and the nonspecific factor of therapeutic alliance. These variables are assessed using (1) the EDE at midtreatment; (2) items from the self-report form of the EDE, the Eating Disorder Examination-Questionnaire (EDE-Q) [42,43], which is administered to patients every week (nine items on binge eating and eating disorder psychopathology); and (3) the Working Alliance Inventory-Short Version (WAI-S) [44,45], which is administered to patients at the end of each CBT session and weekly in the GSH-I condition. Therapeutic alliance proved to be a common ingredient of all psychotherapeutic interventions and to be at least modestly correlated with outcomes in the treatment of many disorders, including bulimia nervosa [46,47]. However, almost nothing is known about how the therapeutic relationship operates online.

The continuous application of the session reports will allow examination of the process of change during treatment. We expect no differential course of these factors between the two treatment conditions.

### Cost-effectiveness

In an associated project, the cost-effectiveness of treatment is being assessed by calculating the direct and indirect costs of both treatment conditions prior, during, and after therapy. By using the Client Sociodemographic and Service Receipt Inventory (CSSRI) [48], the overall health resource utilization of participants as well as productivity losses will be operationalized. To estimate costs, these quantities will then be valued on the basis of market prices. If market prices are not available, administrative prices or mean costs will be used to estimate so-called “shadow prices.”

### Sample size calculations

To test for noninferiority of the GSH-I intervention toward CBT regarding the number of days with OBE episodes over the past 28 days, we specified a noninferiority margin of 1 day in favor of CBT. For sample size calculations, we followed a conservative statistical approach. Assuming a standard deviation of 2.1 days in both groups for the number of binge eating days over the evaluation period (which lies within the range of those observed in previous clinical trials), a sample size of at least 70 participants per group was required for the confirmatory analysis to guarantee statistical power of 80% when testing for noninferiority by applying a two-sample *t*-test. Allowing for a dropout rate of 20% of study participants from T0 to T2, 175 participants need to be recruited overall.

### Randomization

Individuals who met the respective inclusion criteria and who gave their written informed consent to participate were randomized. There were no stratification criteria. To ensure the concealment of allocation, randomization was performed centrally by fax by the Coordination Center for Clinical Trials (KKS) in Marburg. Eligibility assessment, obtaining informed consents, and enrolling the participants in the study were done at the respective study centers.

### Blinding

Treatment and assessment were separated. Therapists and coaches are not involved in assessing treatment outcome, and assessors are not allowed to hold treatment sessions or write e-mails. The statistician who will conduct the statistical analyses was not involved in randomization. Treatment allocation is not disclosed to the statistician until all data checks are completed.

### Statistical analysis

The statistical analysis of the primary end point (number of days with OBE episodes within the past 28 days) will follow a two-step approach. First, a confirmatory analysis of the difference of OBE days from baseline (T0) to the end of treatment (T2) will test for noninferiority of the GSH-I intervention compared to CBT, taking a noninferiority margin of 1 day with OBEs into account. For the noninferiority hypothesis, the per-protocol approach is employed to avoid a bias toward equivalence resulting from intent-to-treat in this specific setting [49]. If the difference of days asymptotically follows a normal distribution, a standard two-sample *t*-test (applying Welch’s correction if different within-group variance appears) will be applied. If the distributional assumption cannot be confirmed on the basis of the observed data, the nonparametric Wilcoxon signed-rank test will be applied.

In a second step, we will carry out an explanatory longitudinal and multivariable regression analysis, taking the absolute number of OBE days within the past 28 days throughout the study from baseline (T0) to follow-up (T3 and T4) into account, adjusting for possible nonspecific predictor variables such as sociodemographic characteristics as well as secondary outcome measures. An advantage of this approach is that we can additionally detect possible moderator and mediator effects influencing treatment outcome [50,51].

As the outcome variable reflects interval count data in a longitudinal setting, we will apply random coefficients modeling of a Poisson regression model [52]. The advantage of this state-of-the-art modeling approach is its ability to incorporate heterogeneity of effects (for example, center-specific or subject-specific) and its tolerance to missing data. Although standard approaches based on relative change rely on complete cases, this longitudinal modeling approach builds up a design matrix with one row for each measurement instead for each patient. A missing outcome at the end of treatment therefore does not necessarily lead to the deletion of the patient from the analysis.

The model, incorporating the log-link as canonical link for the expected value of the Poisson distribution, can be written as follows:

$$\log\left(Y_{\mathrm{ij}}\right)=\theta_{i}+treatment_{i}\cdot time_{j}+x_{i}^{T}\beta +z_{\mathrm{ij}}^{T}\gamma$$

where *Y*_*ij*_ denotes the number of OBE days of patient *i* (*i* = *1*, …, *n*) at time point *j* ∈ (*j* {*0*,*1*,*2*,*3*}); θ_*i*_ is a subject-specific random intercept (following a gamma distribution); *treatment*_*i*_ · *time*_*j*_ is the treatment effect at different time points (where baseline is coded as 0); *z*_*ij*_^*T*^*γ* represents additional subject-specific, time-constant effects (including possible nonspecific predictor variables that were assessed pretreatment, such as age at onset of eating disorder and overweight); and *z*_*ij*_^*T*^*γ* denotes covariates varying over time that might be possible mediators of treatment outcome (considered as the severity of eating disorder and general psychopathology, assessed using the EDE total score and the BDI-II). To account for the possibility of moderator effects, additional interaction terms *treatment*_*i*_ · *z*_*ij*_ can be included for postbaseline measurements (if j ≠ 0). Variable selection will be based on likelihood ratio statistics of nested models.

If the assumption of equal Poisson variation cannot be confirmed and significant over- or underdispersion is present, we consider as an alternative the negative binomial distribution [53] with density:

$$f_{\mathrm{dens}}\left(Y_{\mathrm{ij}}|\mu_{ij,}\sigma \right)=\frac{\Gamma \left(Y_{\mathrm{ij}}+\sigma \right)}{\Gamma \left(Y_{\mathrm{ij}}+1\right)\Gamma \left(\sigma \right)}\cdot \frac{{\left(\frac{\mu_{\mathrm{ij}}}{\sigma }\right)}^{Y_{\mathrm{ij}}}}{{\left(\frac{\mu_{\mathrm{ij}}}{\sigma }+1\right)}^{Y_{\mathrm{ij}}+\sigma }}$$

Here σ is a constant variation parameter, Г(·) expresses the gamma function and log(μ_*ij*_) is modeled by the same additive predictor as described above for the Poisson distribution. If the variation term depends on the time point or other covariates, we will apply generalized additive models for location, scale, and shape (GAMLSS) [54], and we will also model log(σ_*ij*_) with a subject-specific random term [55].

Consistent with previous research [19,41], the analyses will include three categorical outcomes, all determined at posttreatment and follow-up time points: recovered (no OBEs in the past month), improved to subclinical binge eating (less than 4 days with OBEs in the past month), and being at or below a comparative level of eating disorder attitudes and behaviors. The latter rating will be made on the basis of whether the global EDE score is at or below the global EDE score of overweight non-BED treatment-seeking individuals with a sociodemographic profile similar to that of the patients in the current study.

Secondary end points will be analyzed in an explanatory analysis comparing differences between the two treatment groups at T1 and T2, followed by further multivariable regression analyses for cases where differences were detected.

All statistical analysis will be carried out using the open source statistical programming environment R 2.14.2 [56], which provides greater flexibility and far more modeling options than standard commercial software packages.

### Safety aspects

An independent Data Monitoring and Safety Committee (DMSC) was established, which meets once per year. The DMSC is composed of four researchers familiar with the area of the study. The type of information monitored includes patient recruitment, number of dropouts, and all adverse events, including study withdrawals. Any serious adverse events (SAEs) are immediately reported to the PI (MdZ) and the Ethics Committee at the site and are forwarded to the DMSC. All fatal or life-threatening events are defined as SAEs. The DMSC receives recruitment and retention updates on a regular basis from the data center (KKS). The committee prepares a brief report based on the material received, which includes recommendations to the PIs as to whether the continuation of the study is justifiable in view of the number and degree of reported SAEs and the recruitment and follow-up rates are sufficient to guarantee the necessary statistical power.

### Data management

Case report forms (CRFs) were developed using scannable forms, and a study database was set up. All pseudonymized data from patients are stored at the Coordinating Center for Clinical Trials (KKS) in Marburg. Data quality management includes automatic data checks, queries, query handling, and audit trail. All study-related CRFs will be stored for 10 years in the archives of the respective trial site.

### Quality control/monitoring

As an instrument for quality control and quality assurance, the clinical trial is monitored. Monitoring is performed by a psychologist (FS) who has attended a university-based course on good clinical practice (ICH-GCP) and has attained a certificate for monitoring clinical studies. She is supervised by the KKS at the University of Marburg. Monitoring is performed according to the standard operating procedures (SOPs) from the KKS at the University of Marburg. Each trial site receives a minimum of three monitoring visits, including an initiation visit and a close-out visit. The purposes of the monitoring are to verify that the rights of the participants are protected; that the reported trial data are accurate, complete, and verifiable in the source documents; and that the conduct of the trial follows ICH GCP criteria, the study protocol, and its amendments. For every on-site visit, a monitoring report is written and reviewed by the KKS.

### Ethical considerations

The study uses rigorous methodology as required by the funding agency. It is conducted in accordance with ICH GCP and CONSORT criteria. A corresponding declaration had to be submitted comprising the assurance that the trial will be conducted in accordance with the principles of ICH GCP and that the medical institution of the PI of the study will assume the sponsor’s responsibilities in accordance with chapter 5 of ICH GCP. In addition, the study is being carried out in accordance with the latest version of the Declaration of Helsinki.

The final study protocol and the final version of the written informed consent form were approved by the Ethics Committee of the Medical School of the University of Erlangen-Nuremberg, the site where the PI was at the time of the beginning of the study (Ref. No. 4081). Approval from the Ethics Committee was obtained prior to patient enrollment at all trial sites. All protocol modifications are submitted to each Ethics Committee for approval before implementation.

## Results and discussion

INTERBED is the first study comparing GSH-I with standard face-to-face CBT in patients with BED. Self-help interventions and, most recently, internet-based self-help interventions have been shown to be effective in the treatment of BED; however, direct comparisons with CBT are missing.

Furthermore, although CBT is the best-established intervention for BED, until now there have been no controlled multicenter trials conducted in German-speaking countries that have shown the efficacy of this intervention. As BED is still a recent diagnostic category, many cases likely remain undiagnosed, and a large number of patients either receive delayed treatment or never get adequate treatment. A multicenter efficacy trial will advance the dissemination of this evidence-based gold standard treatment in Germany.

Perkins *et al*. [57] summarized in the Cochrane review that, at present, RCTs on self-help in eating disorders are mostly manual-based treatments in book format. Other media for delivering self-help need to be explored as well: “In particular, approaches using new technologies such as the Internet need to be researched further, as they are likely to be more interactive and hence may be more attractive to patients than manual-based approaches” [57]. The German Society for the Internet in Medicine recommends that new technologies such as the internet should be researched further. According to the German Federal Statistical Office, mobile phones, computers, and the internet have become integral parts of our society [58]. Currently, 75% of German adults are using the internet at home or at the workplace [59].

## Conclusions

The detailed methodology for the INTERBED study has been presented. The INTERBED study tries to exclude numerous methodological shortcomings from the outset. In conclusion, the INTERBED study will help to clarify the short- and long-term efficacy of internet-based self-help in comparison to an international gold standard therapy in the treatment of BED. The study will contribute to the dissemination of evidence-based treatment options of BED in Germany. INTERBED will contribute to the specification of how to match patients to treatment.

The study was conducted in accordance with GCP and CONSORT criteria and followed the ethical principles described in the current revision of the Declaration of Helsinki, thus applying highest methodological and ethical standards to a multisite psychotherapy trial that includes face-to-face as well as internet-based treatment approaches.

## Competing interests

TL is director of NetUnion, a provider of health management software. The other authors declare that they have no competing interests related to the content of the article.

## Authors' contributions

MdZ is the principal investigator and grant holder of the INTERBED study and is the coordinator of the Eating Disorders Diagnostic and Treatment Network (EDNET) [60]. AH is the co-principal investigator of the INTERBED study and is responsible for the CBT arm of the study. MdZ and AH designed the study. MdZ, AH, SH, SZ, BTC and HCF are the local principal investigators. OG and AM are the leading biostatisticians of the study. TL provides the technical support for the internet program. CSB is responsible for data management and quality. FS is responsible for the overall coordination of the study and for study monitoring. FS, CSB and OG made substantial contributions to the preparation and methodology of the study protocol. MdZ and AH wrote the first draft and the final draft of the manuscript. All authors critically revised the manuscript for intellectual content. All authors read and approved the final manuscript.
